# Supplementary figures and images for: Maternal inflammation has a profound effect on cortical interneuron development in a stage and subtype-specific manner
Source: Mol Psychiatry. 2019 Oct 8;25(10):2313–29. doi: 10.1038/s41380-019-0539-5 (PMC7515848; doi:10.1038/s41380-019-0539-5)

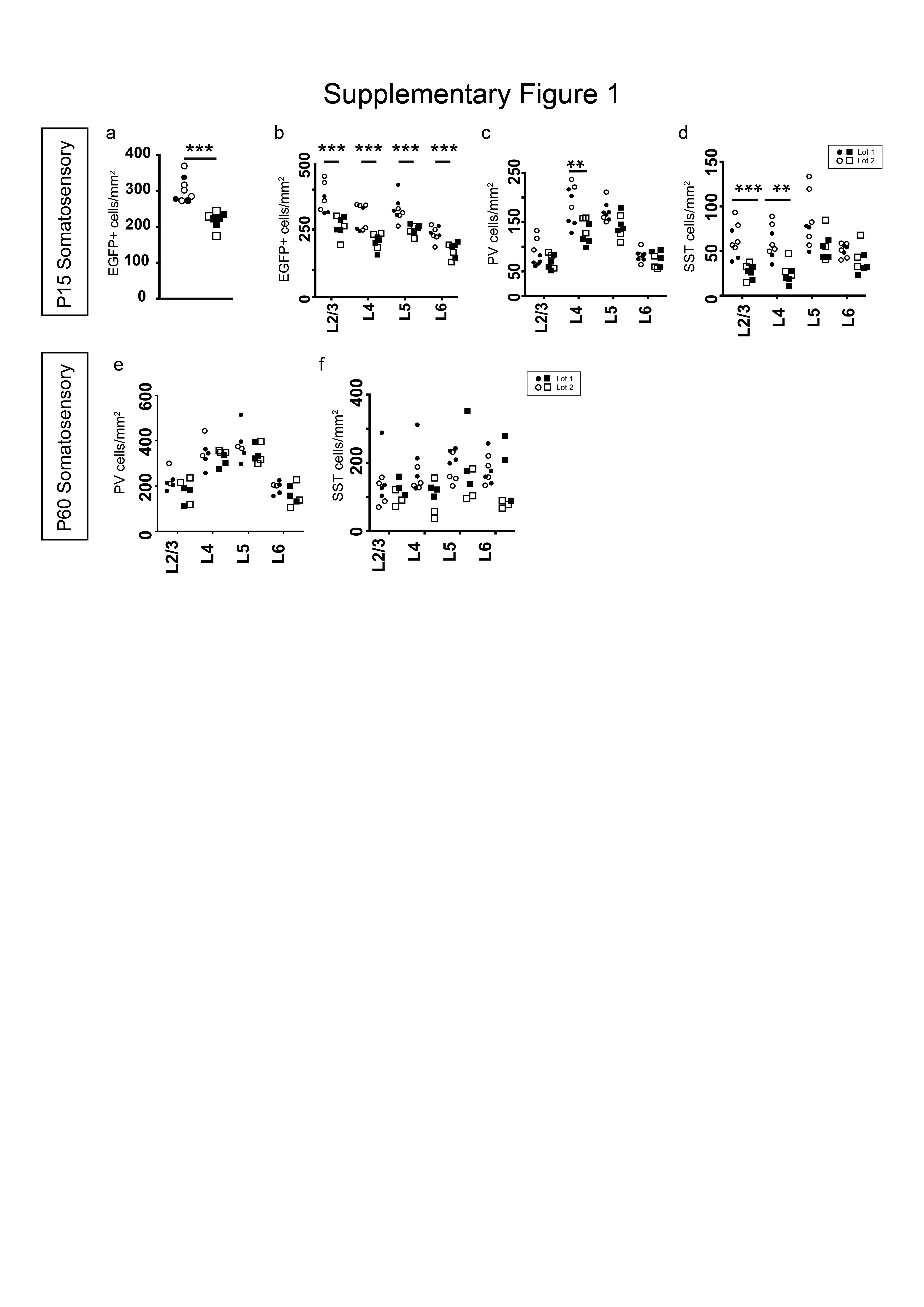

Supplement: Supplementary file 2 — Suppl. Figure 1 [file 41380_2019_539_MOESM2_ESM.jpg]

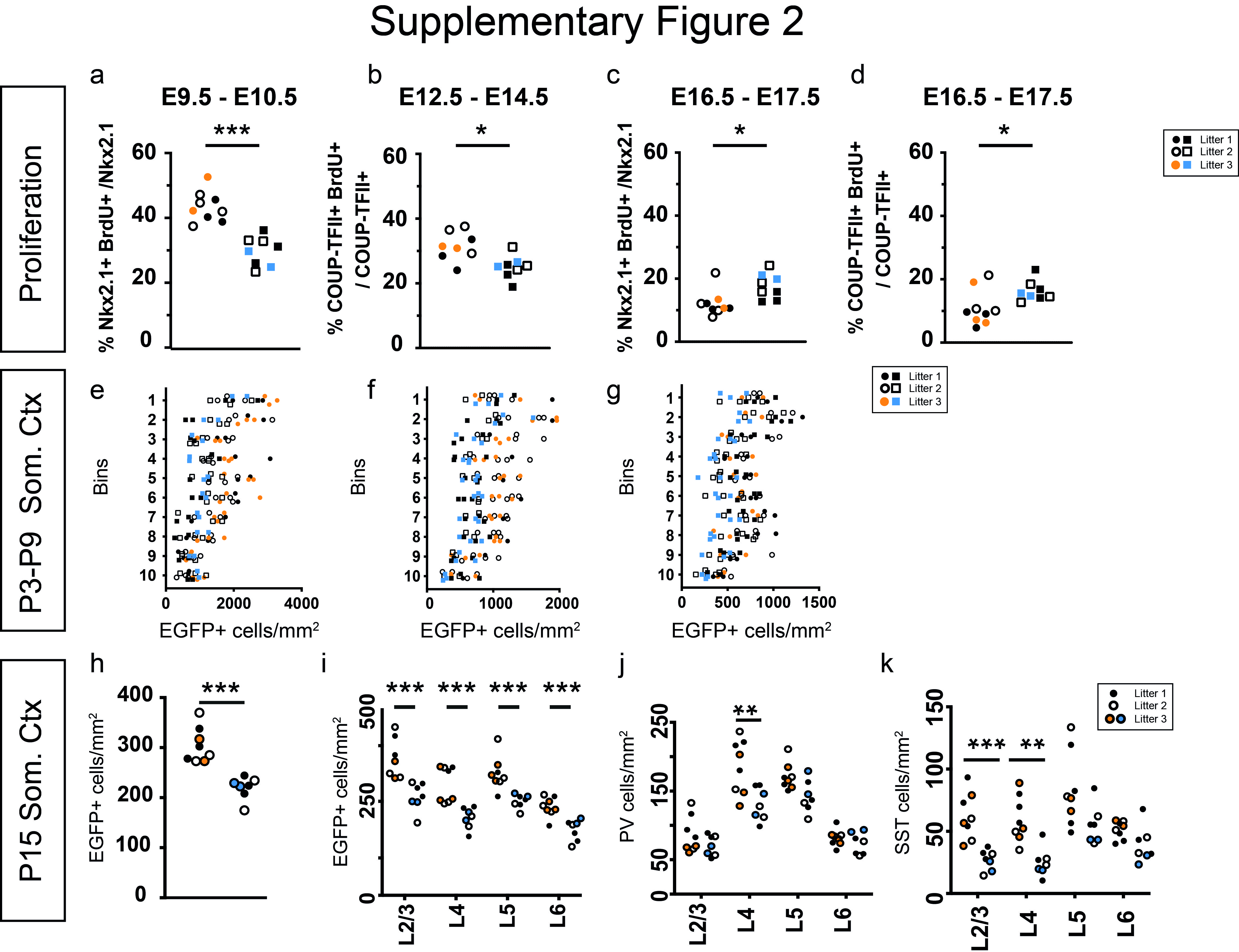

Supplement: Supplementary file 3 — Suppl. Figure 2 [file 41380_2019_539_MOESM3_ESM.jpg]

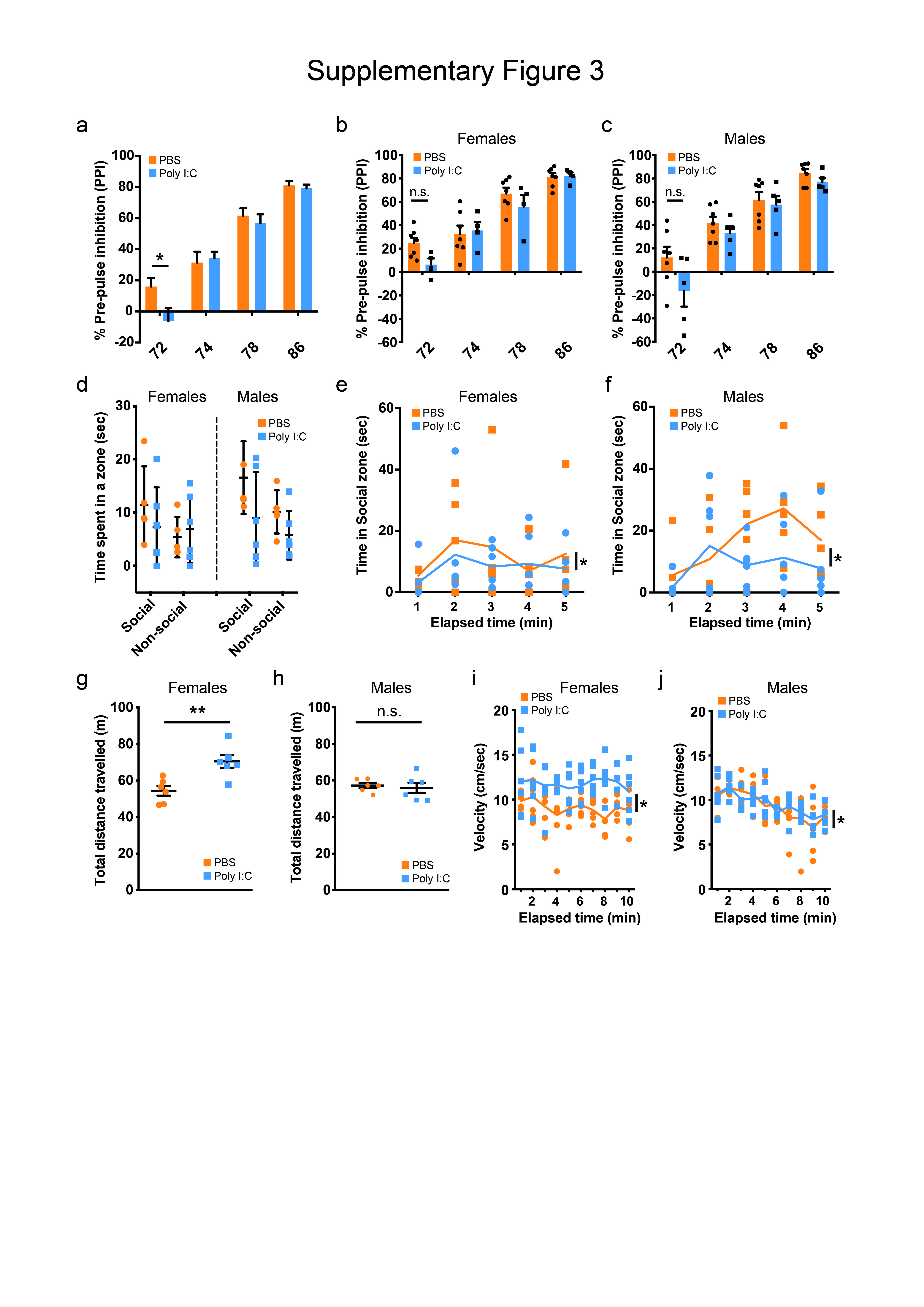

Supplement: Supplementary file 4 — Suppl. Figure 3 [file 41380_2019_539_MOESM4_ESM.jpg]

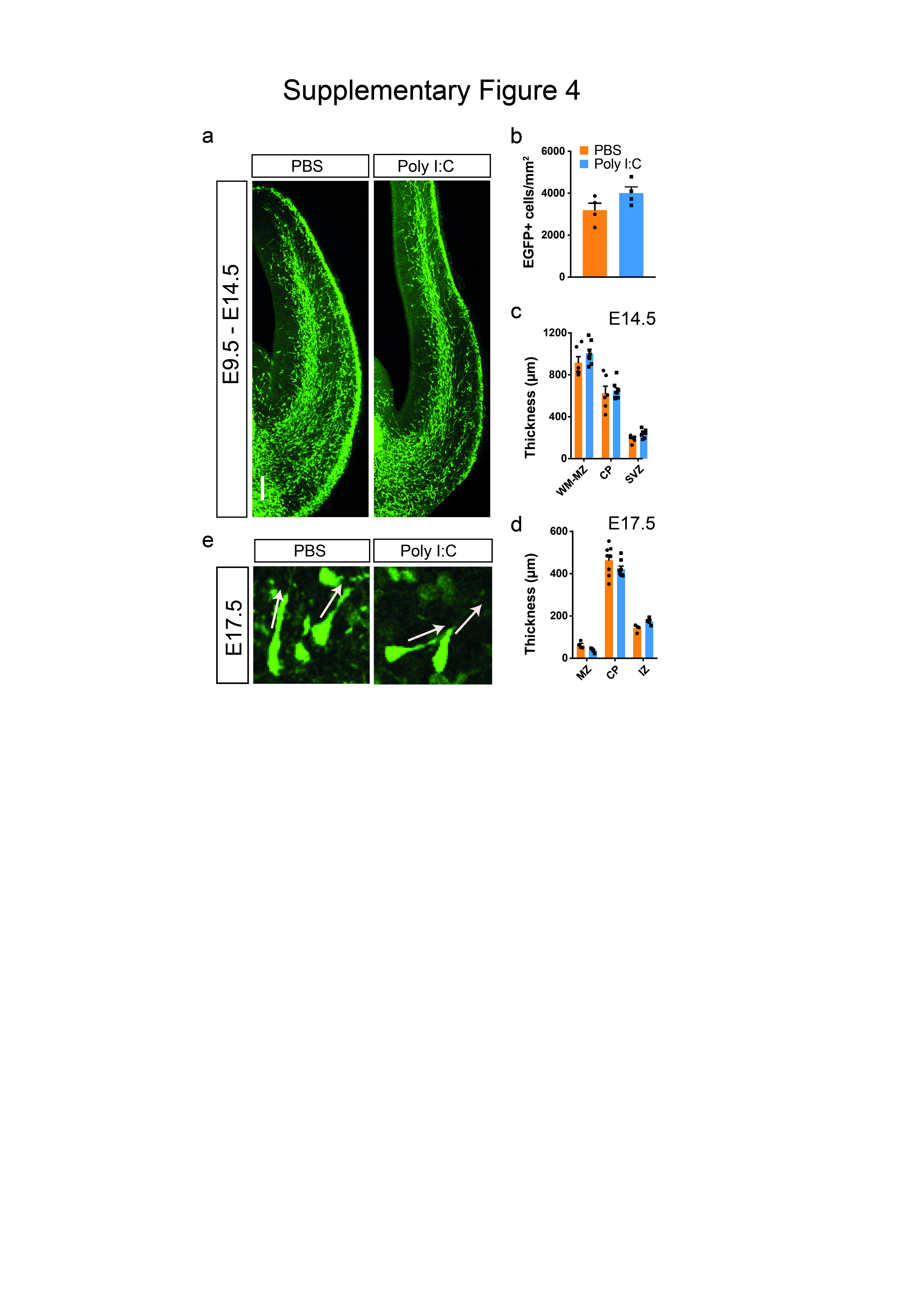

Supplement: Supplementary file 5 — Suppl. Figure 4 [file 41380_2019_539_MOESM5_ESM.jpg]

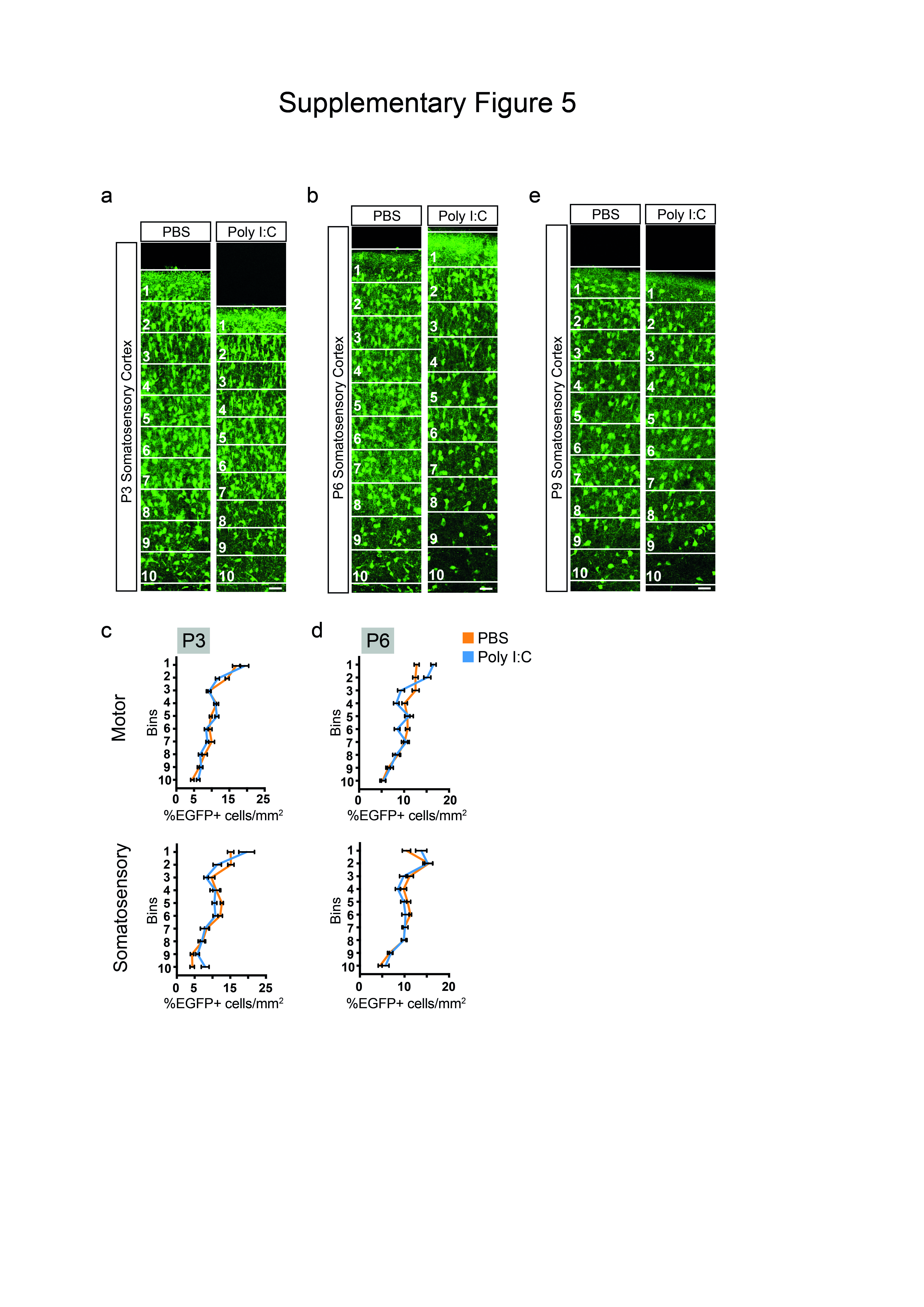

Supplement: Supplementary file 6 — Suppl. Figure 5 [file 41380_2019_539_MOESM6_ESM.jpg]

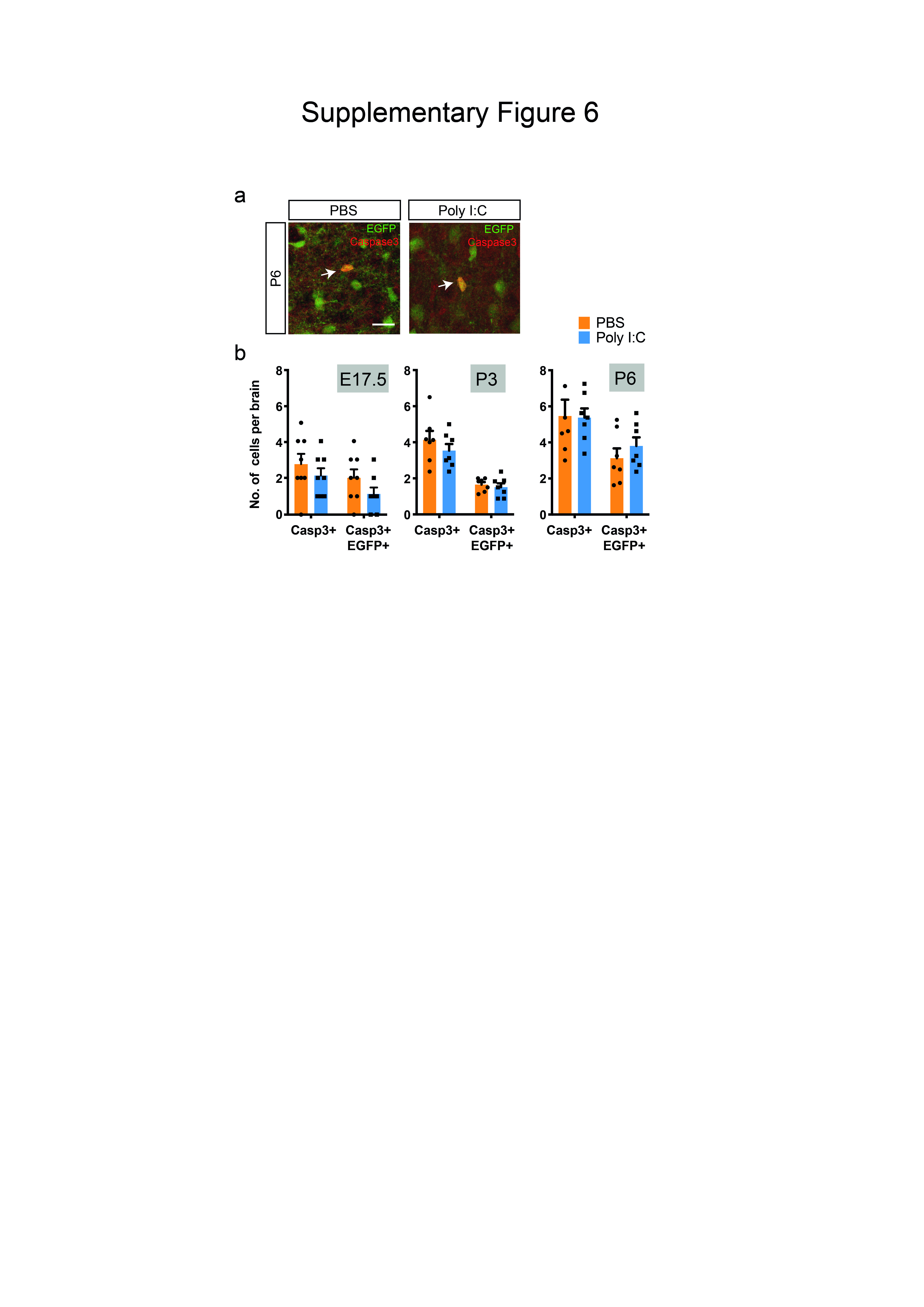

Supplement: Supplementary file 7 — Suppl. Figure 6 [file 41380_2019_539_MOESM7_ESM.jpg]

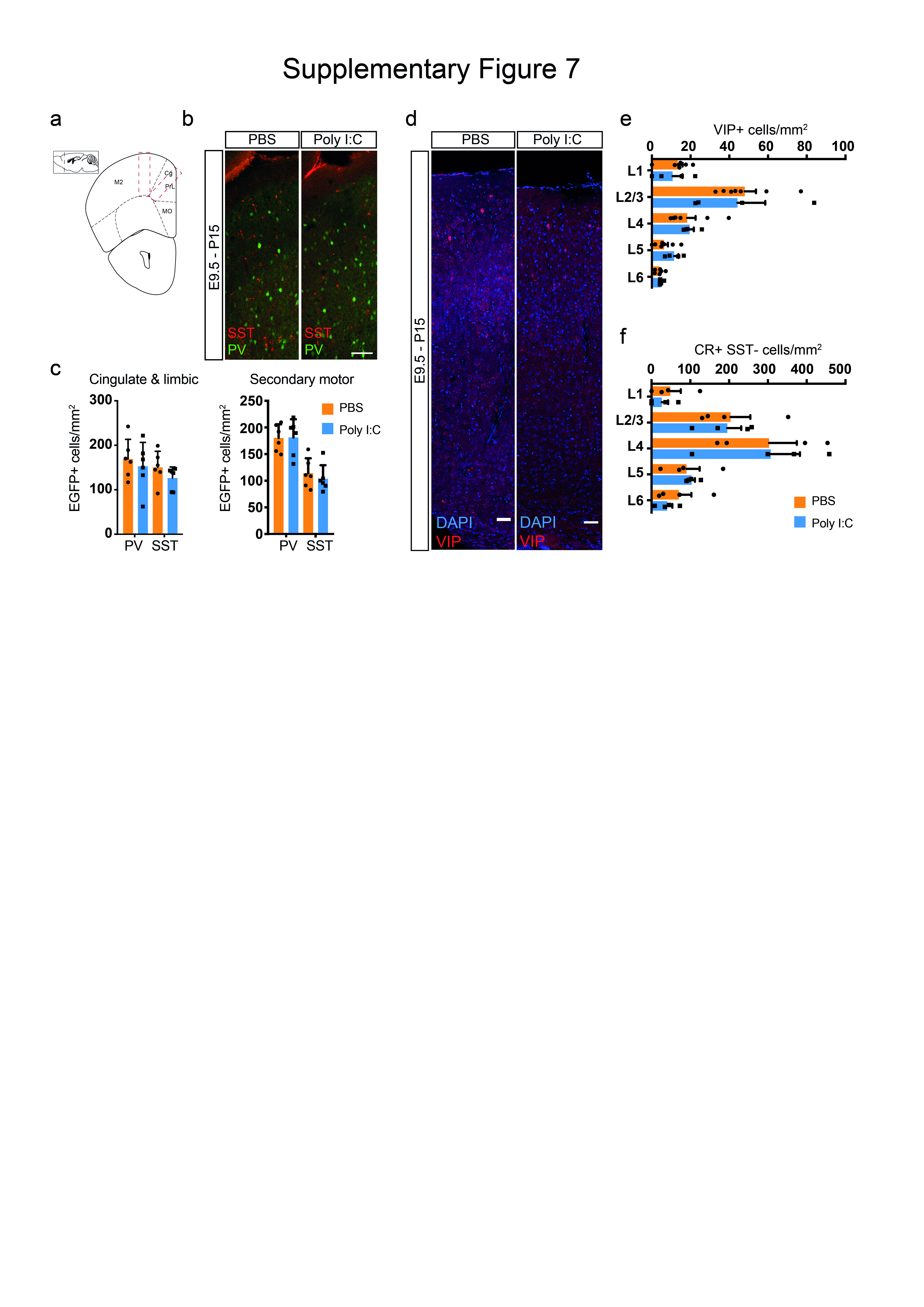

Supplement: Supplementary file 8 — Suppl. Figure 7 [file 41380_2019_539_MOESM8_ESM.jpg]

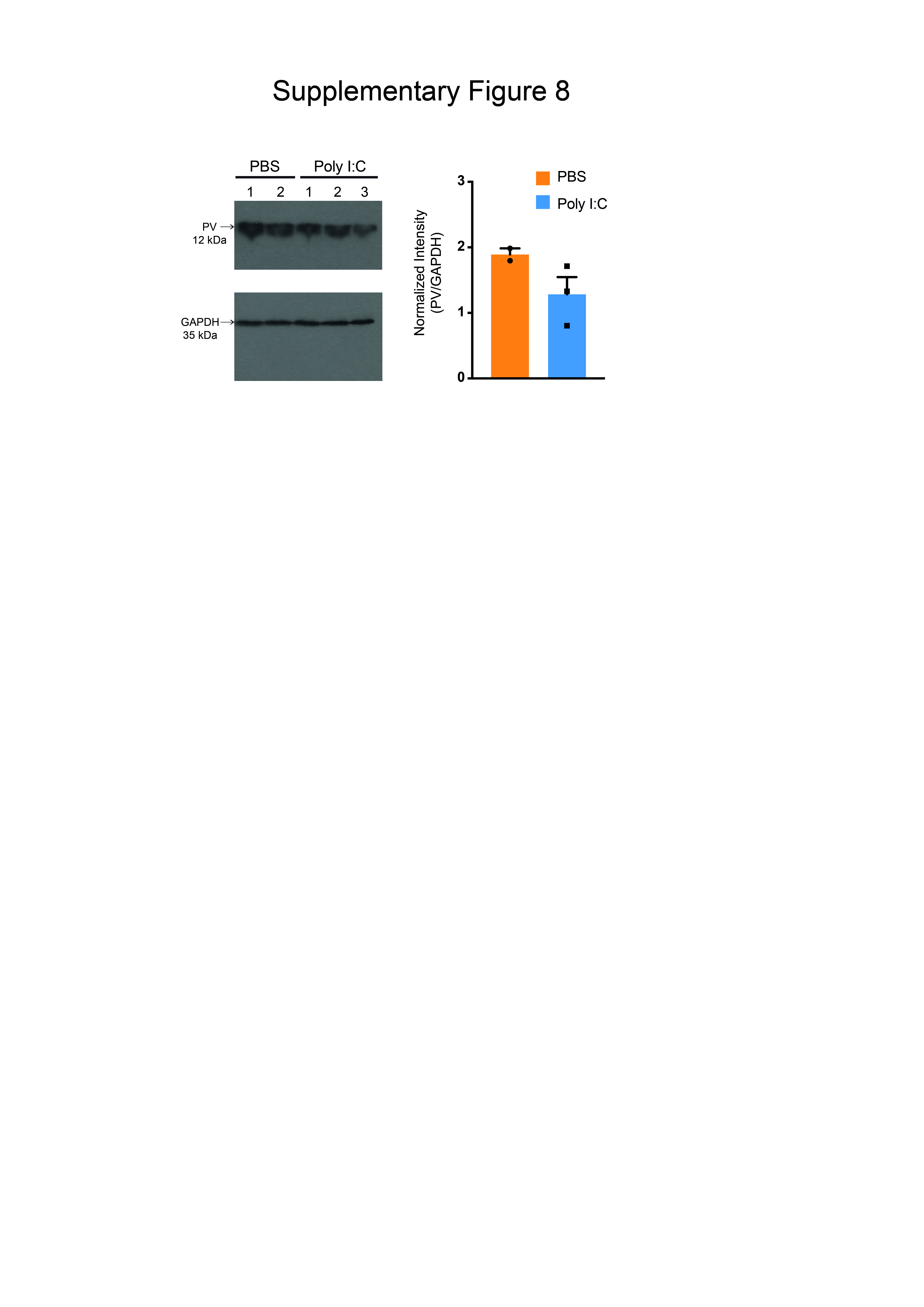

Supplement: Supplementary file 9 — Suppl. Figure 8 [file 41380_2019_539_MOESM9_ESM.jpg]

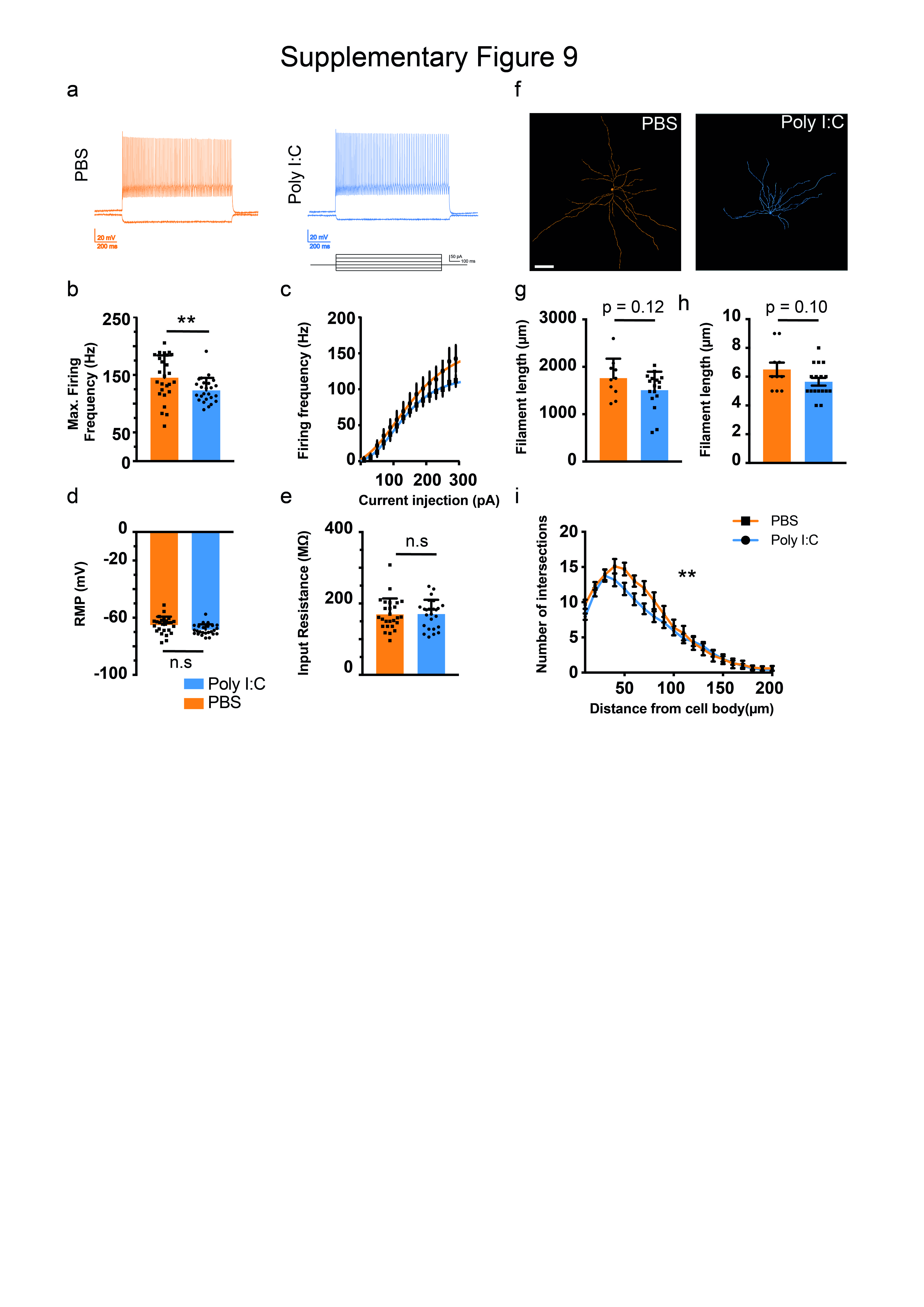

Supplement: Supplementary file 10 — Suppl. Figure 9 [file 41380_2019_539_MOESM10_ESM.jpg]

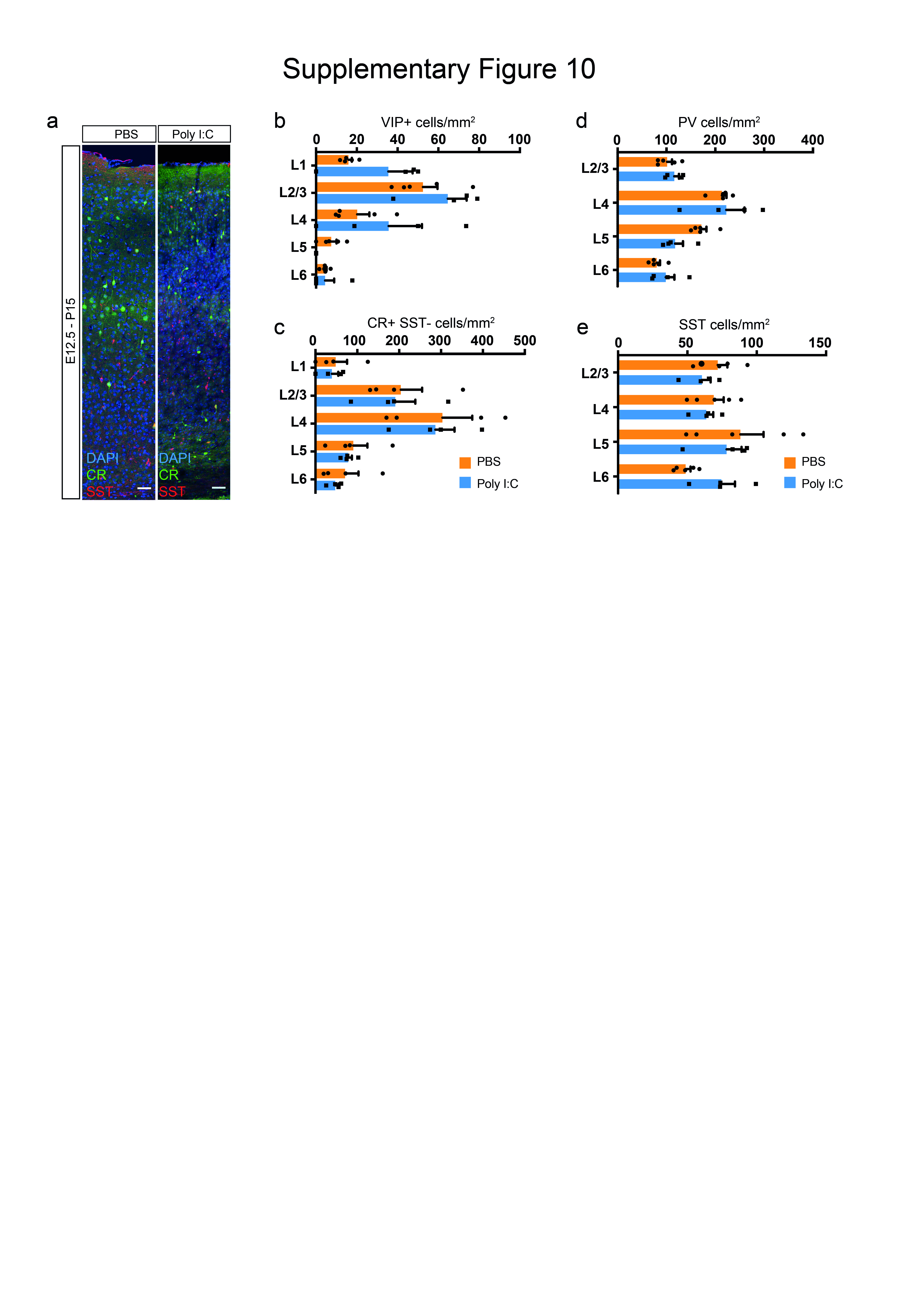

Supplement: Supplementary file 11 — Suppl. Figure 10 [file 41380_2019_539_MOESM11_ESM.jpg]

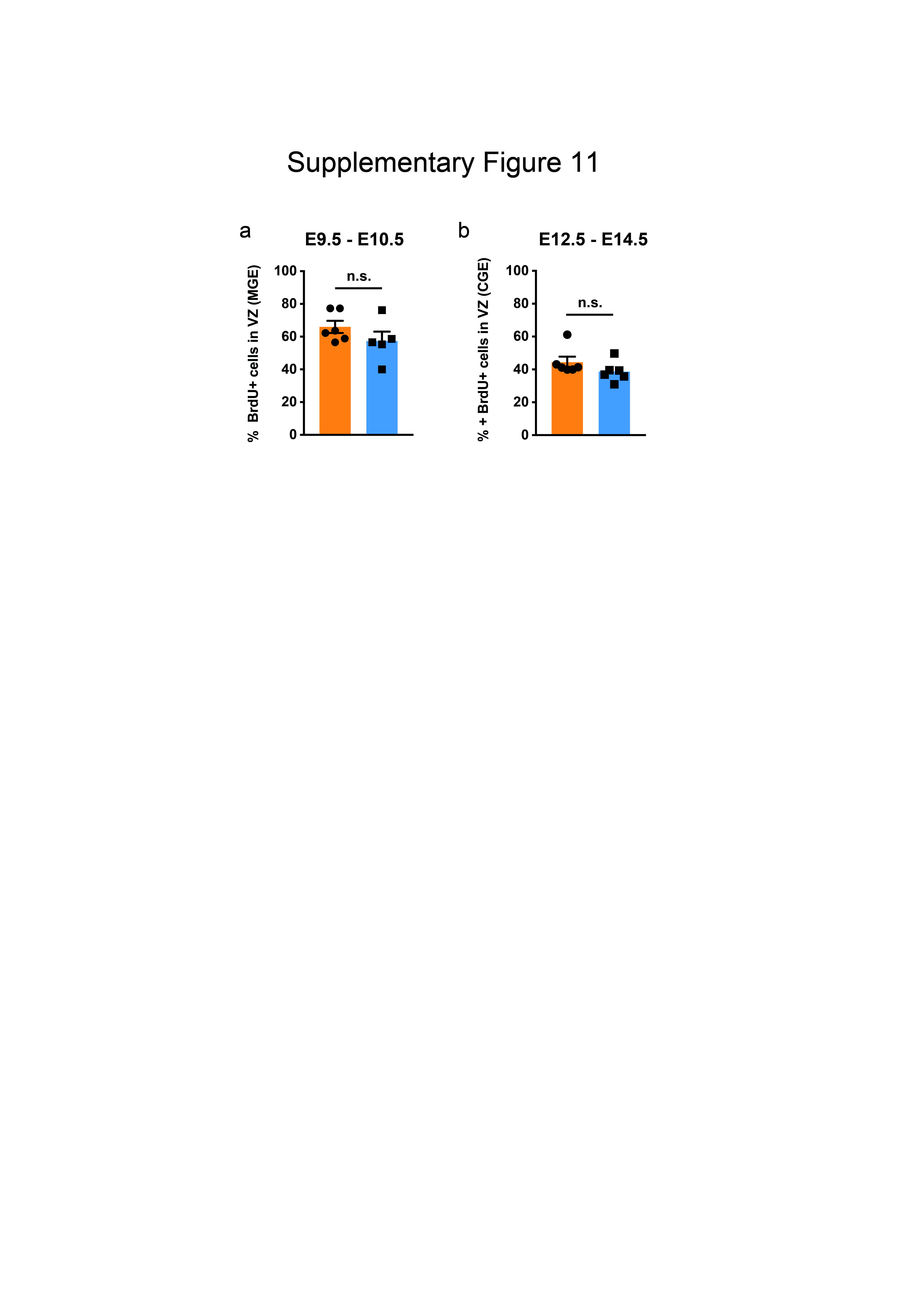

Supplement: Supplementary file 12 — Suppl. Figure 11 [file 41380_2019_539_MOESM12_ESM.jpg]
